# STAT3 suppresses the AMPKα/ULK1‐dependent induction of autophagy in glioblastoma cells

**DOI:** 10.1111/jcmm.17421

**Published:** 2022-06-06

**Authors:** Sujoy Bhattacharya, Jinggang Yin, Chuanhe Yang, Yinan Wang, Michelle Sims, Lawrence M. Pfeffer, Edward Chaum

**Affiliations:** ^1^ Department of Ophthalmology and Visual Sciences Vanderbilt University Medical Center Nashville Tennessee USA; ^2^ Department of Pathology and Laboratory Medicine, The Center for Cancer Research, College of Medicine University of Tennessee Health Science Center Memphis Tennessee USA

**Keywords:** apoptosis, Atg14, Beclin1, Caspase‐3, cathepsin D, LC3‐I/LC3‐II; Prom1/CD133, MRT68921, RAD001 (Everolimus), Sequestome/p62; autophagy flux; mTORC1/2, tuberin/TSC2

## Abstract

Despite advances in molecular characterization, glioblastoma (GBM) remains the most common and lethal brain tumour with high mortality rates in both paediatric and adult patients. The signal transducer and activator of transcription 3 (STAT3) is an important oncogenic driver of GBM. Although STAT3 reportedly plays a role in autophagy of some cells, its role in cancer cell autophagy remains unclear. In this study, we found Serine‐727 and Tyrosine‐705 phosphorylation of STAT3 was constitutive in GBM cell lines. Tyrosine phosphorylation of STAT3 in GBM cells suppresses autophagy, whereas knockout (KO) of STAT3 increases ULK1 gene expression, increases TSC2‐AMPKα‐ULK1 signalling, and increases lysosomal Cathepsin D processing, leading to the stimulation of autophagy. Rescue of STAT3‐KO cells by the enforced expression of wild‐type (WT) STAT3 reverses these pathways and inhibits autophagy. Conversely, expression of Y705F‐ and S727A‐STAT3 phosphorylation deficient mutants in STAT3‐KO cells did not suppress autophagy. Inhibition of ULK1 activity (by treatment with MRT68921) or its expression (by siRNA knockdown) in STAT3‐KO cells inhibits autophagy and sensitizes cells to apoptosis. Taken together, our findings suggest that serine and tyrosine phosphorylation of STAT3 play critical roles in STAT3‐dependent autophagy in GBM, and thus are potential targets to treat GBM.

## 1 INTRODUCTION

Glioblastoma (GBM) is a highly aggressive and lethal brain tumour with poor prognosis.^1^ Despite recent advancements in the molecular characterization of GBM, the median survival of patients is only between 10 and 15 months.^2^ GBM presents major treatment challenges due to its therapeutic resistance and limited drug accessibility, in part due to the blood–brain barrier.^3^ There is a compelling clinical need for a deeper mechanistic understanding of GBM to develop new and targeted approaches to treatment.

Although GBM is characterized by marked intra‐tumoral heterogeneity at both cellular and molecular levels, the PTEN/PI3K/Akt/mTOR signalling axis plays a major role in GBM biology.^4^, ^5^ In addition, accumulating evidence has shown that signal transducer and activator of transcription 3 (STAT3) is an important oncogenic driver in many cancers including GBM.^6^, ^7^ Under normal physiological conditions, cytoplasmic STAT3 undergoes phosphorylation at Tyrosine (Y)‐705 and Serine (S)‐727 residues. STAT3 tyrosine phosphorylation induces homodimerization and/or heterodimerization with other STAT family proteins, nuclear translocation, and DNA binding, leading to the induction of cytokine responsive genes^8^ and anti‐apoptotic genes.^9^ The role of S727 phosphorylation is less well understood, but studies suggest that it may be required for STAT3's maximum transcriptional activity.^10^ STAT3 is constitutively phosphorylated in GBM cancer stem cells (GSCs) and inhibiting STAT3 phosphorylation attenuates GSC‐driven tumour growth,^11^, ^12^ showing that STAT3 plays a critical role in GBM tumorigenesis.

Autophagy is a highly conserved cellular catabolic process that recycles damaged organelles, protein aggregates, and other toxic intracellular debris. Autophagy has a complex and context‐dependent role in tumour development and cancer therapy.^13^ Although autophagy suppresses primary tumour growth, it is required for advanced tumour growth with elevated metabolic demand and promotes multiple steps in tumorigenesis.^14^ Constitutive activation of mTOR signalling impairs basal autophagy in GBM, which enhances proliferation and pluripotency of GSCs.^15^ Conversely, restoration of autophagy through mTOR inhibition reduces the invasive potential of GSCs, suggesting that mTOR hyperactivation sustains GSC metabolism through suppressing autophagy.^16^ Increased autophagy has been associated with both tumour survival and chemoresistance in GBM.^17^

Although these studies underscore the relevance of autophagy in GBM, little is known about the function of STAT3 signalling in regulating autophagy in GBM. Pharmacologic inhibition of either JAK2 (using SAR317461)^18^ or STAT3 (using AG490)^19^ stimulates autophagy in GBM cells. Nuclear STAT3 inhibits autophagy by upregulating anti‐autophagy genes and downregulating pro‐autophagy genes.^20^ An inverse correlation between phosphorylated STAT3 and a stimulator of autophagy, Beclin1, has also been observed in GBM.^21^ Based on these observations, we sought to define the specific STAT3‐dependent signalling mechanisms that modulate autophagy and thereby, impact GBM tumorigenesis and chemosensitivity. Utilizing CRISPR/Cas9 STAT3 knockout (STAT3‐KO) in GBM cells and STAT3‐KO cells restored with wild‐type (WT) STAT3 or with phosphorylation‐defective STAT3 mutants, we demonstrate that STAT3 activation suppresses autophagy, which may be exploitable therapeutically.

## 2 MATERIALS AND METHODS

### 2.1 Reagents

Materials purchased include the following: Foetal bovine serum (Atlanta Biologicals); Enhanced chemiluminescence (ECL) Western blot detection system (Perkin Elmer, Inc.); Protease/Phosphatase Inhibitor Cocktail, cleaved active caspase‐3 (Asp 175); LC3‐I/LC3‐II; SQSTM1/p62, p‐Akt Ser473, HDAC‐6, phospho‐S6 Ribosomal protein Ser235/236, p‐STAT3 Y705, p‐STAT3 Ser727, Acetyl‐STAT3 Lys685, total‐STAT3, p‐AMPKα Thr172, Total‐ΑΜPΚα, p‐ULK1 Ser555, p‐ULK1 Ser638, Total‐ULK1, p‐TSC2 Ser1387, p‐TSC2 Ser1462, Total‐Tuberin/TSC2, Beclin‐1, BNIP3 and Cathepsin‐D antibodies (Cell Signalling Technology, Inc.); Alexa‐Fluor 488 conjugated, Alexa‐Fluor 647, and Cy3 conjugated secondary antibodies (Molecular Probes); Anti‐trimethyl STAT3 Lys180, and Bafilomycin A1, (EMD Biosciences/Millipore Corp.); ULK1 Inhibitor (MRT68921) (MedChemExpress). The ULK1 siRNA and transfection reagent were obtained from Santa Cruz Biotechnology. The cell death detection ELISA kit was purchased from Roche (Millipore Sigma). All chemicals were of the highest purity commercially available.

### 2.2 Cell culture

MT330 (UTHSC, Department of Neurosurgery) and LN229 (ATCC CRL‐2611) were grown in DMEM containing high glucose, containing 10% foetal bovine serum, and supplemented with 1X antibiotic‐antimycotic solution (Gibco, Thermo Fisher Scientific) at 37°C with 5% CO_2_, as described previously.^22^

STAT3 was knocked out in both LN229 and MT330 cells by CRISPR/Cas9‐mediated gene editing, and the constructs for wild‐type (WT), Y705‐STAT3 and S727A‐STAT3 mutants were expressed in STAT3‐KO cells by lentiviral transduction, as described previously.^12^, ^23^

### 2.3 Western blotting

Cell lysates were prepared using mammalian protein extraction buffer (Cell Signalling Technology) and a protease inhibitor cocktail followed by SDS‐PAGE. Proteins were transferred to Immobilon‐P membranes (Millipore Bedford) and probed with primary antibodies overnight at 4°C in TBS buffer containing 0.1% Tween‐20 and 5% nonfat dry milk (Bio‐Rad). Membranes were subsequently incubated with horseradish peroxidase‐conjugated secondary antibodies at room temperature for 1 h, and the immunocomplexes were visualized by the ECL detection system (Perkin Elmer) quantified on the Azure Biosystems C500. Membranes were stripped and re‐probed for actin or GAPDH as loading controls. Representative Western blots from three experiments are shown. Densitometric analysis of all Western blots was performed using Image J software.

### 2.4 Immunoprecipitation

Glioblastoma cells were rinsed with ice cold PBS and lysed using a cell lysis buffer (Cell Signalling Technology, Inc.) containing protease and phosphatase inhibitors (Thermo Fisher Scientific). The lysates were clarified by centrifugation at 14,300 *g* for 15 min at 4°C. The cell extracts containing equal amounts of protein were incubated with STAT3 antibody overnight at 4°C followed by addition of protein A/G agarose beads (Santa Cruz Biotechnology) with gentle rocking for 2 h. The beads were washed 3 times with lysis buffer and once with PBS, and the immunocomplexes were released by heating in Laemmli sample buffer and analysed by Western blotting using trimethyl‐STAT3 antibody (EMD Biosciences/Millipore Corp.).

### 2.5 Immunofluorescence and confocal microscopy

Cells were cultured in chamber slides (Millipore) to ~70% confluence and washed with PBS. Cells were fixed in 4% paraformaldehyde and methanol, and permeabilized with 1% Triton X‐100. After blocking with 5% goat serum, cells were incubated with anti‐rabbit LC3 and anti‐mouse p62 antibodies and subsequently stained with Alexa Fluor 488 (goat anti‐rabbit) and Alexa Fluor 633 (goat anti‐mouse) secondary antibodies, as described previously.^24^ DNA was counterstained with Vectashield mounting media with DAPI (Vectra Laboratories). Images were captured on a Zeiss LSM700 laser scanning confocal microscope.

### 2.6 siRNA transfection

MT330 cells were grown to 60%–70% confluency in 6‐well tissue culture plates, and siRNA transfection was performed using a protocol available from Santa Cruz Biotechnology. For ULK1 siRNA transfection, the cell monolayer was washed with siRNA transfection medium (Santa Cruz) and the siRNA/transfection reagent mixture was added dropwise on to the cell monolayer and incubated overnight at 37°C in a CO_2_ incubator. The following day complete growth medium containing 2 times the normal serum and antibiotics was added without removing the transfection mixture. After an additional incubation for 18–24 h, the medium was aspirated and replaced with fresh 1X growth medium. After another 24 h of incubation, cells were treated with or without 100 nM Baf and assayed for autophagy and apoptosis markers. Efficiency of transfection was monitored using FITC‐conjugated control siRNA.

### 2.7 Real‐time quantitative PCR

TRIzol reagent (Thermo Fisher Scientific) was used to extract total‐RNA. Total RNA concentrations were quantified by measuring A260 and A280 using NanoDrop spectrophotometry. Total‐RNA (1 mg) was reverse‐transcribed to cDNA using a kit from Promega and following manufacturer's instructions. The cDNA was diluted 1:5 with DNase‐free water. Real‐time qPCR was performed using an Ariamx Real‐Time PCR system (Agilent Technologies) with 2.5 ml of the cDNA product in a 25 ml reaction mixture containing 1X SYBR® Green Master Mix (Applied Biosystems) and 120 nM forward and reverse primers. The primers used for human ULK1 forward (5′‐ GGGCAAGTTCGAGTTCTCC‐ 3′), reverse (5′‐ GCCATTTCCTGGAAGTCGTA‐ 3′); BNIP3 forward (5′‐ CGCAGCTGAAGCACATCC‐ 3′), reverse (5′ – CTTGGAGCTACTCCGTCCAG– 3′); p62 forward (5′‐GCCTCTGGTTCTGACACTTT‐3′), reverse (5′‐GGTGAGGTGGAAGGCATTTA‐3); beta actin forward (5′‐ ACCTTCTACAATGAGCTGCG‐ 3′) and reverse (5′‐ CCTGGATAGCAACGTACATGG‐ 3′) sequences were used. The qPCR conditions were 50°C for 2 min, 95°C for 10 min, followed by 40 cycles of 95°C for 15 s and 60°C for 1 min, as described previously. Samples were analysed using the comparative ΔCT method, and gene expression was normalized to beta‐actin expression. Data were presented as fold changes with empty‐vector as control set to 1.0.

### 2.8 Apoptosis

The quantitative DNA fragmentation assay was performed using a cell death ELISA kit as described earlier.^25^ Briefly, MT330 cells were either treated with MRT68921 or ULK1 siRNA and the attached cells were washed twice with Dulbecco's phosphate buffered saline (DPBS). Cells were lysed, and an aliquot of the nuclei‐free supernatant was placed in streptavidin‐coated plates and incubated with anti‐histone biotin and anti‐DNA peroxidase‐conjugated antibodies for 2 h at room temperature. After incubation, the samples were aspirated, and the wells were washed 3 times with incubation buffer. After the final wash, 100 μl of the substrate, 2,2′‐azino‐di[3‐ethylbenzthiazolin‐sulfonate], was added in the wells for 3 min at room temperature. The absorbance was read at 405 nm using the SpectraMax iD3 microplate reader (Molecular Devices). Results were expressed as absorbance at 405 nm/mg protein/min.

### 2.9 Statistical analysis

All data were analysed by GraphPad Prism 9 program (GraphPad Software Inc.), and an unpaired 2‐tailed Student's *t*‐test was used to assess statistical significance. Data are expressed as mean ± SE. Experiments were repeated three times, with triplicate samples for each. Unless otherwise stated, values of *p < 0.05, **p < 0.01, ***p < 0.001 and ****p < 0.0001 were considered significant.

## 3 RESULTS

### 3.1 STAT3 deletion increases autophagy

**FIGURE 1:**
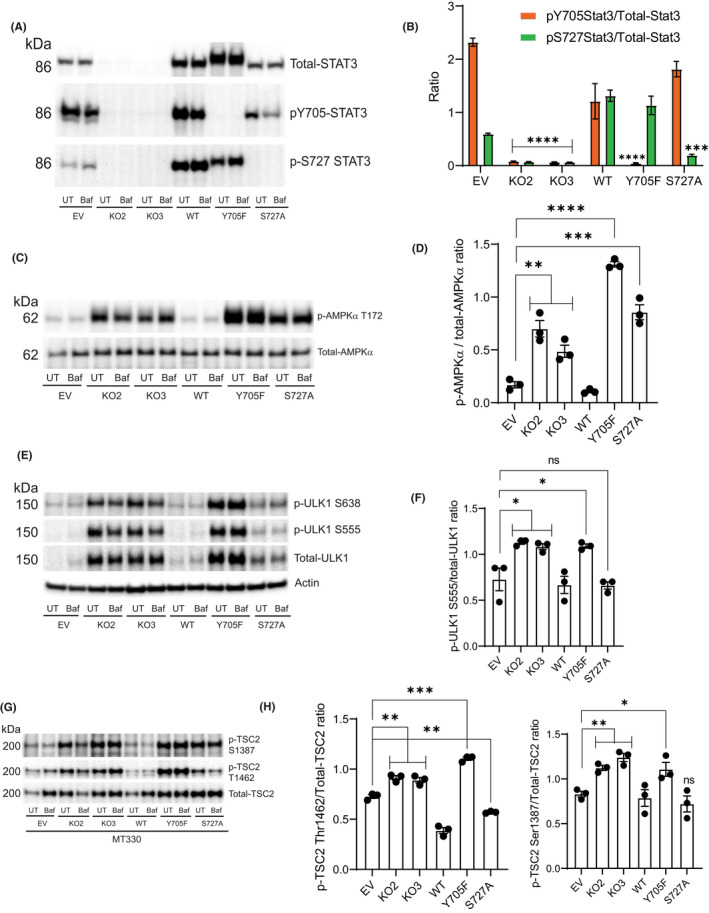
STAT3 deletion activates autophagy through AMPKα‐ULK1‐TSC2 signalling pathways in MT330 cells. (A) Confluent EV MT330 cells, STAT3‐knockout cell line # 2 (KO2), STAT3‐knockout cell line # 3 (KO3), STAT3‐KO3 rescued with wild‐type (WT), and STAT3‐KO3 cells expressing Y705F‐STAT3 and S727A‐STAT3 mutants were exposed to Bafilomycin (Baf, 100 nM) for 3 h. Untreated (UT) cells served as controls. Total cell lysates were prepared and immunoblotted with indicated antibodies. (B) Quantification of the ratio of phospho‐STAT3,, and total‐STAT3 from three independent experiments. (C) Cell lysates were analysed for p‐AMPKα T172. Blots were stripped and probed for total‐AMPKα. (D) Quantification of the ratio of phosphorylated and total AMPKα shown in C. (E) Cell lysates were analysed for p‐ULK1 S555 and S638. Blots were stripped and probed for total‐ULK1. (F) Quantification of the ratio of phospho‐ULK1 S555 and total‐ULK1. (G) Western blotting of cell lysates with phospho‐TSC2 antibodies. Blots were stripped and probed with total‐TSC2 antibody. (H) Quantification of the ratio of phospho‐T1462‐ and total‐TSC2 and phospho‐S1387 and total‐TSC2

We previously established STAT3‐KO MT330 GBM cells by CRISPR/Cas9 gene editing; as a control, cells were transduced with empty vector (EV). Absence of STAT3 protein in MT330 cells was validated in whole‐cell extracts by immunoblotting with antibodies to STAT3 (Figure 1A). STAT3 expression was restored in the STAT3 knockout cells by transduction with lentiviral vectors encoding either the WT‐STAT3 or the STAT3 mutants (Y705F and S727A). In EV‐transduced MT330 cells, STAT3 was phosphorylated on both Y705, and Ser727 (Figure 1A,B). While STAT3‐KO completely abolished STAT3 serine and tyrosine phosphorylation, rescue of KO cells with WT‐STAT3 restored these phosphorylation events. Expression of the Y705F mutant in KO cells restored S727 phosphorylation but showed no Y705 phosphorylation (Figure 1B). Similarly, expression of the S727A mutant restored Y705 phosphorylation but S727 phosphorylation was blocked (Figure 1B).

To characterize whether STAT3‐deficiency may regulate autophagy in MT330 GBM cells, we examined AMPKα and Unc‐51‐like kinase 1 (ULK1) phosphorylation status in STAT3‐KO MT330 cells. AMPKα is activated in response to cellular stress^26^ and activates autophagy either through ULK1 or by impairing mTOR‐dependent inhibition of ULK1.^26^, ^27^ Deletion of STAT3 in MT330 cells significantly increased AMPKα activity, as determined by the ratio of phosphorylated AMPKα (assessed by Thr172 phosphorylation in the catalytic domain) to unphosphorylated AMPKα (Figure 1C,D). Rescue of STAT3‐KO cells with WT‐STAT3 blocked AMPKα activation. Expression of the Y705F and S727A mutants in STAT3‐KO cells had significantly higher levels of AMPKα activation compared to STAT3‐KO cells (Figure 1C,D). Since AMPKα activates autophagy through ULK1 phosphorylation at multiple sites, we next assessed ULK1 phosphorylation at S555 and S638 residues (Figure 1E,F). STAT3 deletion in MT330 cells markedly increased total ULK1 protein expression and phosphorylation at all sites (Figure 1E). Our results demonstrate significantly higher levels of phosphorylated ULK1 S555 in STAT3‐KO cells compared to EV (Figure 1F). Given that ULK1 acts as a direct target for both mTORC1 and AMPKα,^28^ these data suggest that ULK1 is a critical mediator of autophagy in GBM cells. Expression of the Y705F mutant in KO cells showed a similar effect on phosphorylated ULK1 S555. However, cells expressing the S727A mutant had no significant effect on phosphorylated ULK1 (Figure 1F) suggesting that S727‐STAT3 phosphorylation is involved in AMPKα‐dependent pathway but does not involve ULK1 to suppress autophagy. Reconstitution with WT‐STAT3 in KO cells completely reversed both pathways. Bafilomycin (Baf) treatment had no effect on AMPKα and ULK1 phosphorylation in MT330 cells, indicating that these upstream pro‐autophagy pathways are unaltered by disruption of autophagy flux.

Since AMPKα inhibits mTORC1 by phosphorylating and activating TSC2,^29^ we examined TSC2 phosphorylation in STAT3‐KO MT330 cells. We found that KO of STAT3 significantly increased TSC2 S1387 and Thr1462 phosphorylation, which was reversed with WT‐STAT3 expression (Figure 1G,H). Expression of Y705F mutant also increased TSC2 S1387 and T1462 phosphorylation. In contrast, cells expressing the S727A mutant showed significantly lower levels of phosphorylated TSC2 T1462 but high levels of S1387 phosphorylation, demonstrating that STAT3 S727 phosphorylation selectively regulates TSC2 T1462 phosphorylation (Figure 1G,H). Thus, AMPKα activation in STAT3‐KO cells acts through more than one pathway to activate autophagy, by directly activating ULK1 and by indirectly impairing inhibition of ULK1 through activation of TSC2 S1387 phosphorylation. Since Akt inhibits TSC2 via phosphorylation on T1462, resulting in basal mTORC1 activation, it is possible that mTORC1 could phosphorylate and inhibit ULK1. This may be counteracted by ULK1 phosphorylation at S555 leading to its sustained activity, which is required for maintaining autophagy flux in STAT3‐KO cells.

### 3.2 STAT3‐deletion increases autophagic flux in LN229 cells

**FIGURE 2:**
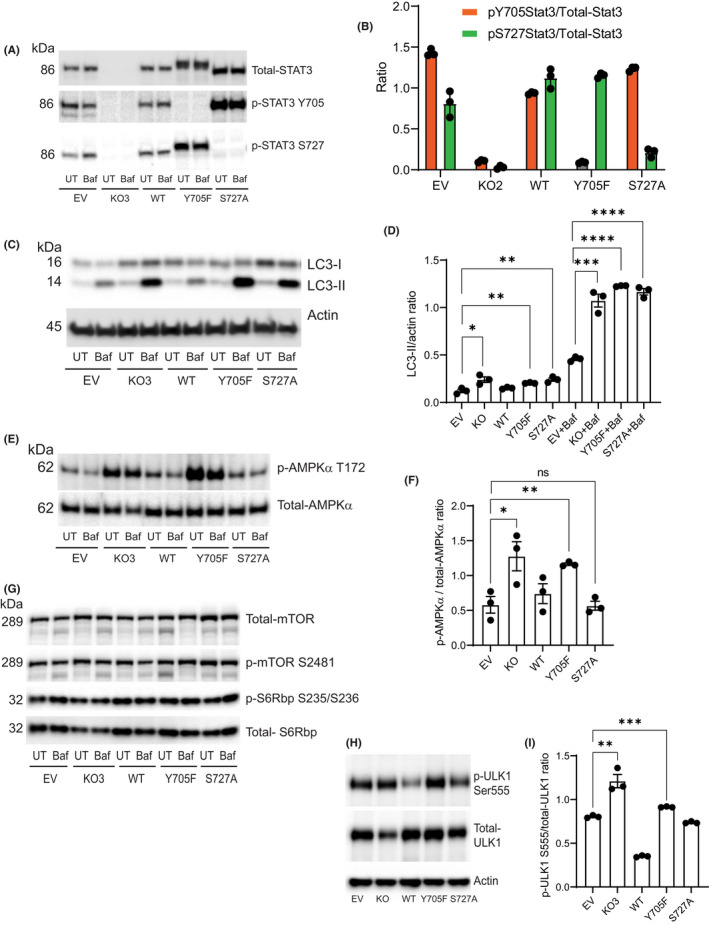
STAT3 Represses autophagy in LN229 cells. (A) EV LN229 cells, STAT3‐knockout (KO), STAT3‐KO cells rescued with WT STAT3, and STAT3‐KO cells expressing Y705F‐STAT3 and S727A‐STAT3 phosphorylation‐defective mutants, were exposed to Bafilomycin (Baf, 100 nM) for 3 h or left untreated (UT). Total cell lysates were prepared and immunoblotted with indicated antibodies with β‐Actin serving as a loading control. (B) Densitometric analysis from *n* = 3 observations of the ratio of phosphorylated STAT3 to total‐STAT3 shown in A. (C) EV, STAT3‐KO and STAT3 mutant expressing lines with treated with or without 100 nM bafilomycin for 3 h. Cell lysates were immunoblotted for LC3‐I/II with Actin as a loading control. (D) Quantification of data shown in C. (E) Cell lysates were analysed for p‐AMPKα Thr172. Blots were stripped and probed for total‐AMPKα. (F) Quantification of the ratio of phosphorylated and total AMPKα shown in E. (G) Cell lysates were immunoblotted with the indicated antibodies. (H) Cell lysates were analysed for phospho‐ULK1 S555 and total‐ULK. (I) Quantification of data shown in H

We also examined the role of STAT3 in GBM autophagy regulation in another GBM cell line, LN229 cells, in which STAT3 was also knocked out or restored with WT‐STAT3 and STAT3 phosphodeficient mutants (Y705F and S727A). Consistent with the results in MT330 cells, STAT3 was phosphorylated on both Y705 and S727 sites in EV‐transduced LN229 cells (Figure 2A,B). Rescue with WT‐STAT3 lentivirus in KO cells restored STAT3 expression and STAT3 Y705 and S727 phosphorylation. As with the MT330 studies, expression of the Y705F mutant eliminated Y705 phosphorylation and restored S727 phosphorylation (Figure 2A,B), and expression of the S727A mutant in STAT3‐KO cells restored Y705 phosphorylation but not S727 phosphorylation (Figure 2A,B).

To validate our findings on the role of STAT3 in autophagy, LN229 cells were treated with Baf and LC3‐II levels determined. Baf is a specific inhibitor of vacuolar‐type ATPase that is known to prevent the fusion of autophagosomes with lysosomes resulting in LC3‐II accumulation and blockade of the autophagic flux.^30^ Since autophagy is a dynamic process where microtubule‐associated protein light chain‐I (LC3‐I, precursor) is rapidly converted to lipidated LC3‐II, we examined the relative levels of LC3‐I and LC3‐II in LN229 cells treated with Baf. While basal and Baf treated LN229 cells had low LC3‐II levels, deletion of STAT3 significantly increased LC3‐II levels, demonstrating constitutive autophagy upon STAT3 deletion (Figure 2C,D). Furthermore, rescue with WT STAT3 reduced the LC3‐II levels, confirming that STAT3 suppresses autophagy in LN229 cells. As shown in Figure 2C,D, cells expressing the Y705F and S727A mutants showed high basal and Baf‐induced LC3‐II levels. These results suggest that both STAT3 serine and tyrosine phosphorylation events are necessary for suppressing autophagy in GBM cells, which is entirely consistent with our findings in MT330 cells.

Deletion of STAT3 in LN229 cells increased AMPKα activity (assessed by T172 phosphorylation in the catalytic domain),^31^ without altering total AMPKα protein levels (Figure 2E) indicating higher AMPKα activation (Figure 2F). While expression of the Y705F mutant in STAT3‐KO cells also showed AMPKα activation, expression of S727A mutant did not alter AMPKα phosphorylation, supporting a critical role for Y705 STAT3 phosphorylation in suppressing autophagy.

We also examined the role of STAT3 in mTOR phosphorylation in LN229 cells. The mTOR protein and its basal phosphorylation status at S2481 were unaltered in the KO and lines expressing STAT3 phosphorylation‐inactive mutants (Figure 2G), demonstrating that basal mTOR activity is unaltered. Furthermore, expression of S6 ribosomal protein (S6Rbp) and its phosphorylation at S235/S236 were also unaltered in KO cells and cells expressing STAT3 mutants (Figure 2G). Therefore, autophagy induction in LN229 cells either lacking STAT3 or expressing the phosphorylation‐defective STAT3 mutants involves AMPKα but is independent of the mTOR pathway.

We examined whether STAT3‐KO activates ULK1 signalling in LN229 cells. Our data show that ULK1 protein was detectable in LN229 cells. Although STAT3‐KO decreased ULK1 protein levels (Figure 2H), it significantly increased ULK1 activity (Figure 2I). Rescue with WT‐STAT3 decreased ULK1 activity (Figure 2H,I), but expression of the Y705 mutant increased ULK1 activity in LN229 cells, which is consistent with our findings in MT330 cells. However, expression of the S727A mutant in STAT3‐KO cells had no effect on ULK1 activity, also suggesting that the Y705 mutant is the key critical regulator of AMPKα and ULK1 signalling in LN229 cells.

### 3.3 The dependence of autophagy on STAT3

**FIGURE 3:**
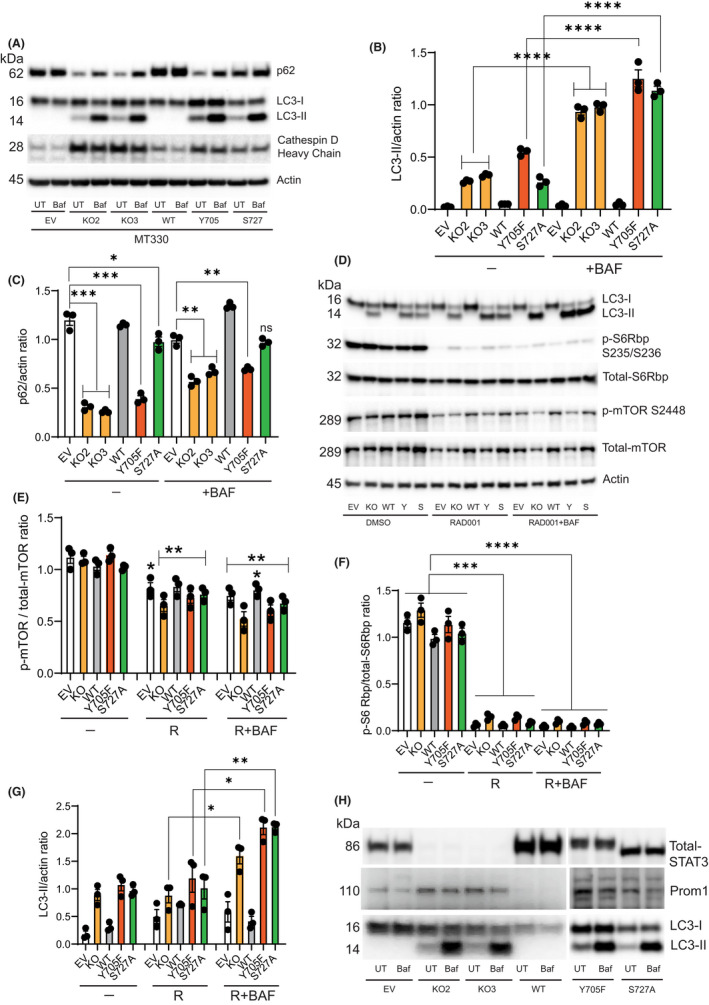
STAT3‐Ko activates autophagy through mTOR‐independent but Prom1‐dependent signalling pathways in MT330 cells. (A) EV MT330 cells, STAT3‐KO, STAT3‐KO rescued with WT‐STAT3, and STAT3‐KO cells expressing Y705F (Y) and S727A (S) STAT3 mutants were treated with or without 100 nM bafilomycin for 3 h. Cell lysates were analysed for LC3‐I/II, p62 and CathepsinD. Quantification of (B) LC3‐II/Actin ratio and (C) p62/Actin ratio. (D) DMSO, Everolimus (RAD001) (10 μM) and RAD001 (10 μM) + Baf (100 nM) for 3 h. Cell lysates were immunoblotted with the indicated antibodies and beta actin was used as an internal loading control. (E) Quantification of phospho‐mTOR S2448 and total‐mTOR ratio. (F) Quantification of phospho‐S6 Ribosomal protein (Rbp) S235/236 and total‐S6 Rbp ratio. (G) Quantification of LC3‐II/Actin ratio presented in D. (H) EV, STAT3‐KO cells, KO3 rescued with WT‐STAT3, and KO3 cells expressing Y705F and S727A mutants were grown to confluence and treated with Baf (100 nM) for 3 h. Cell lysates were analysed by Western blotting using antibodies specific for STAT3, Prom1 and LC3‐I/II

To further assess the impact of STAT3 deletion and rescue of KO cells with STAT3 mutants, MT330 cells were treated with Baf. Under basal conditions, LC3‐II levels were undetectable in control MT330 EV cells and Baf treatment had no effect on LC3‐II levels, showing minimal basal autophagy (Figure 3A,B). In contrast, STAT3 deletion in MT330 cells significantly increased basal LC3‐II, which was further increased by Baf treatment. Rescue with WT STAT3 ablated basal and Baf‐induced LC3‐II levels (Figure 3A,B). These results demonstrate that KO of STAT3 enhances LC3‐II levels and autophagy flux, which were reversed by WT‐STAT3 restoration. Expression of Y705F and S727A phosphorylation‐defective mutants also resulted in high basal and Baf‐induced LC3‐II levels as observed in STAT3‐KO cells, providing further evidence for the role of these phosphorylation events in STAT3 autophagy suppression. Together, these results show that loss of STAT3 function enhances LC3‐II lipidation and autophagic flux through activation of the AMPKα/ULK1/TSC2 signalling axis.

The p62 protein has often been used as an inverse marker for autophagy flux, and high expression of p62 is found in GBM patient tumours.^32^ High p62 levels were observed in control MT330 (EV) cells, but p62 levels were significantly decreased in STAT3‐KO cells consistent with induction of autophagy in these cell lines (Figure 3A,C). Baf treatment partially increased p62 accumulation in STAT3‐KO cells suggesting impaired autophagosomal degradation. Rescue of KO cells with WT‐STAT3 restored p62 accumulation to that of control EV cells demonstrating the role of STAT3 in suppressing autophagy. Expression of Y705F mutant significantly decreased p62 accumulation, which is evidence of increased autophagosomal degradation and re‐activation of autophagy (Figure 3A,C). Similarly, expression of the S727 mutant decreased p62 accumulation, suggesting a decline in its autophagosomal degradation. Conversely, Baf treatment did not significantly change LC3‐II levels in cells expressing the S727A mutant. Our results, therefore, strengthen our hypothesis that Y705 STAT3 plays a key role in suppressing autophagy.

Additional evidence for the induction of autophagy was seen by an increase in the levels of mature heavy chain Cathepsin‐D in STAT3‐KO cells, which was blocked when KO cells were rescued with WT‐STAT3 (Figure 3A). In cells expressing phosphorylation‐defective STAT3 mutants, the levels of mature Cathepsin D were higher compared to control EV cells (Figure 3A). Cathepsins are lysosomal proteases that are essential for the breakdown of the recycled cellular components sequestered by the autophagosomes.^33^ Increase in the levels of lysosomal proteases upon deletion of STAT3 is consistent with autophagosome‐fusion and autophagy activation.

We used a catalytic mTOR inhibitor, Everolimus (RAD001), to further characterize the role of STAT3 in regulating autophagy by mTORC1. Treatment of EV MT330 cells with Everolimus inhibited both mTOR S2448 phosphorylation (Figure 3D,E) and pS6K phosphorylation (a downstream target of mTORC1) (Figure 3D,F) but had no significant effect on autophagy induction (Figure 3D,G). STAT3 deletion had no effect on basal mTOR S2448 and S6Rbp phosphorylation S235/236 (Figure 3D–F) indicating that STAT3 does not regulate mTORC1 activity in MT330 cells. LC3‐II levels in STAT3‐KO cells significantly increased in response to Everolimus and Baf compared to Everolimus alone, demonstrating that combining STAT3 deletion with mTOR inhibition increases autophagy (Figure 3D,G). These effects were reversed in cells reconstituted with WT STAT3. Expression of either Y705F or S727A mutants (as with the STAT3‐KO cells) significantly increased LC3‐II/actin ratio in the presence of Everolimus and Baf (Figure 3D,G). These results demonstrate that Everolimus enhances autophagic flux in cells lacking STAT3 and STAT3‐KO cells expressing STAT3 phosphodeficient mutants.

Since Prom1 is a pro‐autophagy protein in normal cells,^34^ we examined Prom1 expression and its correlation with autophagy induction in GBM cells. STAT3‐KO increased Prom1 expression, which was reversed by rescue with WT‐STAT3 in MT330 cells (Figure 3H). Expression of Y705F and S727 mutants in STAT3‐KO MT330 cells also showed increased Prom1 expression, which correlated with high autophagy (Figure 3H). These results suggest that the induction of autophagy in cells lacking STAT3 is in part dependent on Prom1 expression. Reconstitution of WT‐STAT3 in these cells completely reversed these effects confirming STAT3‐mediated suppression of autophagy.

### 3.4 The role of STAT3 on autophagy‐associated gene expression

**FIGURE 4:**
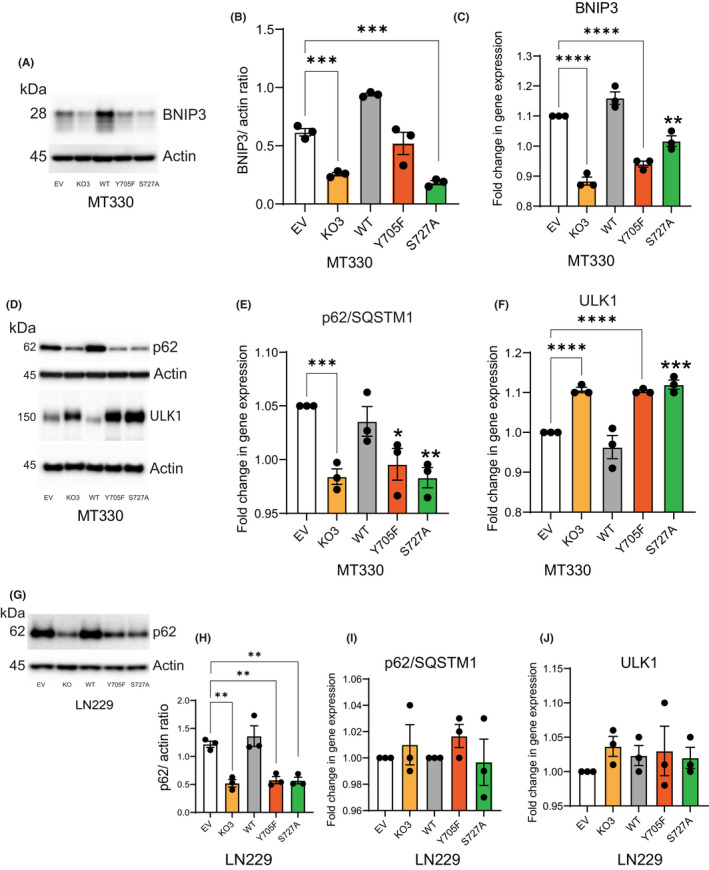
Transcriptional regulation of autophagy‐associated genes. (A) EV MT330 cells, STAT3‐KO and STAT3‐KO cells rescued with WT‐STAT3, or phosphorylation‐defective mutants were analysed for BNIP3 expression by Western blotting. Blot was stripped and probed for actin. (B) Quantification of data shown in A. (C) Fold change in BNIP3 gene expression in MT330 cells. (D) MT330 cells were analysed for p62 and ULK1 protein levels. Blots were stripped and probed for actin. Fold change in (E) p62 and (F) ULK1 genes in MT330 cells. (G) Analysis of p62 protein levels in LN229 cells. Blot was stripped and probed for actin. (H) Quantification of data shown in G. Fold change in (I) p62 and (J) ULK1 genes in LN229 cells

BNIP3 has been shown to play a role in regulating the autophagy pathway.^35^ In previous studies, we showed that STAT3‐KO in MT330 cells inhibited the expression of classical STAT3 genes such as Cyclin D1 and vascular endothelial growth factor.^23^ We found that STAT3‐KO decreases BNIP3 protein levels in MT330 cells (Figure 4A,B). Expression of Y705F and S727A mutants decreased BNIP3 protein levels (Figure 4A) and WT‐STAT3 reversed these changes. Since we found that STAT3‐KO increased ULK1 protein levels and decreased p62 levels in MT330 cells (Figures 1 and 3), we next performed qPCR on these autophagy‐related genes. In agreement with these observations, our data show that STAT3 increases BNIP3 gene expression in MT330 cells (Figure 4C), showing a correlation between mRNA and protein levels. Although BNIP3 is a potential target of ULK1, decreased BNIP3 protein levels in STAT3‐KO and mutant expressing cells indicate that BNIP3 is degraded by ULK1‐dependent autophagy, a mechanism found to decrease BNIP3 protein levels in tumour cells.^36^

Consistent with data in Figure 3, STAT3‐KO reduced p62 protein and transcript levels (Figure 4D,E), which is reversed by WT‐STAT3 expression. In addition, both Y705 and S727 residues are necessary for expression of p62 transcript and protein levels since cells expressing STAT3 mutants showed similar expression of p62 to STAT3‐KO cells. In contrast, STAT3 reduces expression of ULK1 gene (Figure 4F) and STAT3 phosphorylation on both Y705 and S727 residues is necessary for ULK1 repression (Figure 4D,F). Together, these results demonstrate that STAT3 may regulate autophagy in MT330 cells in part through the transcriptional regulation of several autophagy‐related genes.

We next examined p62 and ULK1 transcript levels in LN229 cells. Our data demonstrate that STAT3‐KO and expression of STAT3 mutants significantly reduced p62 protein levels (Figure 4G,H). To demonstrate whether mRNA and protein levels of p62 and ULK1 were correlated, we examined their transcript levels in LN229 cells. We found that p62 and ULK1 gene expressions were unaltered in STAT3‐KO LN229 cells (Figure 4I,J), which differed from their expression in STAT3‐KO MT330 cells. In addition, expression of STAT3 mutants in STAT3‐KO cells had no effect on p62 and ULK1 genes. These results demonstrate that reduction of p62 protein levels in LN229 cells is due to enhanced degradation during autophagy, not due to changes in p62 gene transcription.

### 3.5 Immunolocalization of LC3 and p62

**FIGURE 5:**
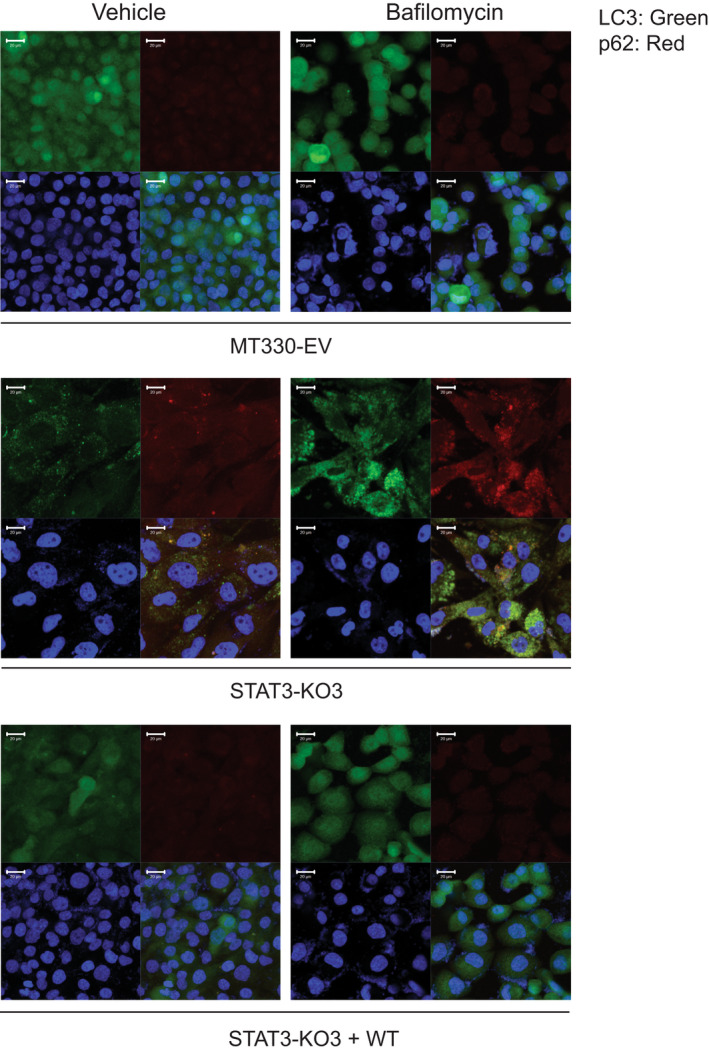
Immunolocalization of LC3 and p62 in MT330 cells. EV MT330 cells, STAT3‐KO, STAT3‐KO cells rescued with WT‐STAT3, were grown on chamber slides, and treated with or without 1μΜ Baf for 48 h. Cells were fixed and immunostained for LC3 (green), p62 (red), and counterstained with DAPI (blue), and analysed by confocal microscopy. Scale bar 20 μM

To further validate the role of STAT3 in autophagy, we performed immunolocalization studies to detect LC3 and p62 puncta formation. Under basal conditions, we observed diffuse LC3 (green) and p62 (red) staining (green) predominantly in the cytoplasm of control (EV) MT330 cells with some nuclear staining (Figure 5). Treatment of EV cells with a low dose of Baf (1μΜ) for 48 h had no effect on LC3 and p62 staining. These observations are consistent with our data presented in Figure 3 and demonstrate impaired autophagy in MT330 cells. Conversely, STAT3‐KO cells without bafilomycin treatment showed distinct LC3 and p62 puncta formation, and treatment with bafilomycin for 48 h increased both LC3+ and p62 puncta formation and their co‐localization (Figure 5). Reconstitution of WT‐STAT3 in STAT3‐deleted cells completely reversed these effects. These observations confirm cellular induction of autophagy flux in response to STAT3 deletion.

### 3.6 Targeting of ULK1 blocks autophagy and induces apoptosis in STAT3‐KO MT330 cells

**FIGURE 6:**
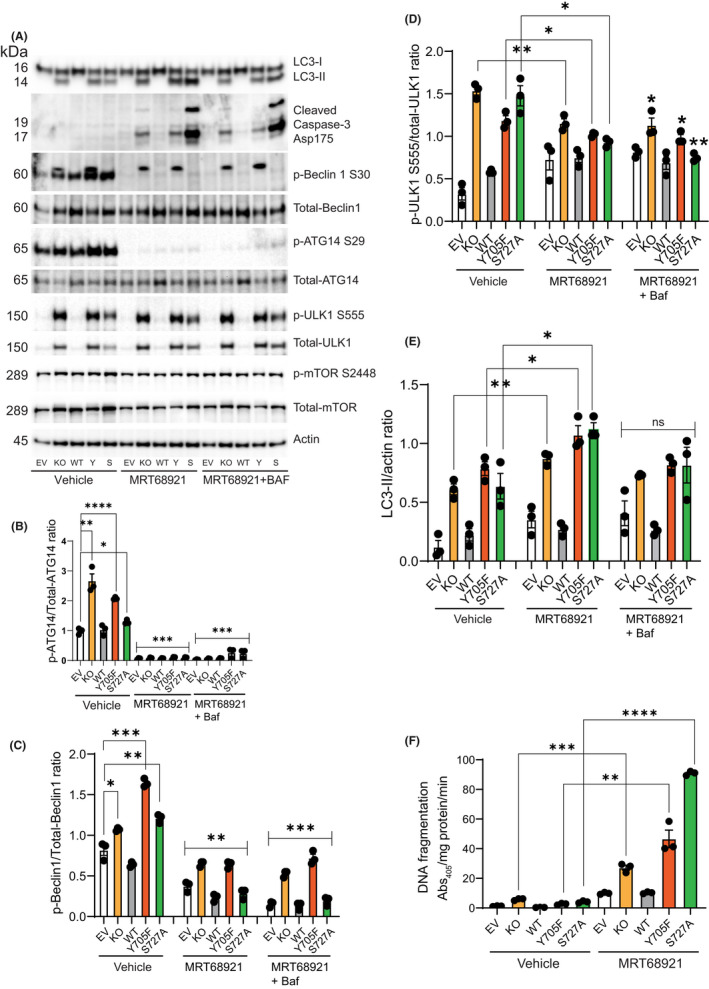
Pharmacologic inhibition of ULK1 activity inhibits autophagy and induces apoptosis in STAT3‐KO MT330 cells. (A) MT330 EV, STAT3‐KO, WT, Y705F‐STAT3 (Y) and S727A‐STAT3 (S) mutant expressing cells were treated with the ULK1 inhibitor, MRT68921 (20 μM), in the presence or absence of Baf (100 nM) for 5 h. Total cell lysates were immunoblotted with indicated antibodies with β‐Actin serving as a loading control. Quantification of the ratio of (B) phospho‐ and total‐ATG14; (C) phospho‐ and total‐Beclin1; (D) phospho‐ and total‐ULK1; (E) LC3‐II and Actin. (F) Apoptotic cell death (DNA fragmentation) was measured by ELISA after treatment of MT330 cells with 20 μΜ MRΤ68921 for 5 h (mean SE, *n* = 3)

Because STAT3‐KO in MT330 cells induces autophagy and concomitantly activates AMPKα/ULK1 signalling, we tested the effects of pharmacologic inhibitors of ULK1. Previous studies showed that MRT68921 (inhibitor of ULK1 and ULK2) blocks autophagy flux,^37^, ^38^ demonstrating the importance of ULK kinase activity in autophagy. Since activated ULK1 phosphorylates both ATG14 on S29 and BECN1 on S30 for autophagy,^39^ we measured ATG14 and BECN1 phosphorylation in response to MRT68921 treatment. We found that STAT3‐KO cells and cells expressing STAT3 mutants had elevated levels of ATG14 S29 phosphorylation and Beclin1 S30 phosphorylation (Figure 6A–C). MRT68921, completely blocked ATG14 S29 phosphorylation demonstrating the efficacy of this inhibitor in targeting ULK1 and showing that constitutive activation of ULK1 in STAT3‐KO and STAT3‐mutant expressing lines leads to increased ATG14 phosphorylation on S29 (Figure 6B). MRT68921 also reduced Beclin1 phosphorylation (Figure 6C), demonstrating that ULK1 regulates Beclin1 activation in STAT3‐KO and STAT3‐mutant expressing lines. Importantly, MRT68921 significantly inhibited the high levels of ULK1 phosphorylation in STAT3‐KO cells as well as cells expressing STAT3 mutants (Figure 6D) confirming that Atg14 and Beclin1 are downstream targets of ULK1 signalling in MT330 cells. MRT68921 did not significantly block mTOR S2448 phosphorylation (Figure 6A). Since mTORC1 is phosphorylated predominantly on S2448, these observations suggest that ULK1 inhibition has no significant effect on mTORC1.

MRT68921 also significantly increased LC3‐II levels in STAT3‐KO cells and in STAT3‐KO cells transduced with the phosphorylation‐defective Y705F or S727A mutants (Figure 6A,E). Given that ULK1 inhibition has been shown to disrupt autophagosome maturation downstream of LC3 conjugation,^37^ the increase in LC3‐II levels in MRT68921‐treated cells reflects a decrease of LC3 autolysosomal degradation. Treatment of cells with MRT68921 and Baf did not increase LC3‐II levels as compared to DMSO‐treated controls, showing inhibition of autophagy flux. Taken together, these results strongly suggest that MRT68921 blocks autophagy flux in cells lacking functional STAT3.

To further elucidate the role of ULK1 inhibition in MT330 cells, we determined the effect of MRT68921 treatment on apoptosis as determined by caspase‐3 cleavage. MRT68921 induced apoptosis in STAT3‐KO and STAT3‐mutant expressing lines but not in control EV and cells reconstituted with WT‐STAT3 (Figure 6A). Interestingly, STAT3‐KO cells expressing the S727A‐STAT3 mutant showed high levels of active caspase‐3 suggesting increased a critical role of Y705 STAT3 phosphorylation in the sensitivity of GBM cells to MRT68921‐induced apoptosis. To confirm apoptosis induction by MRT68921, we used a highly sensitive ELISA to quantify apoptotic cell death. MRT68921 significantly increased apoptosis in STAT3‐KO and mutant expressing cells when compared to control cells (Figure 6F). The extent of apoptotic cell death was considerably higher in cells expressing the S727A‐STAT3 mutant (Figure 6F), which is consistent with the levels of active caspase 3 in response to MRT68921 treatment (Figure 6A). These observations demonstrate that autophagy can have a cytoprotective role in STAT3‐KO and STAT3‐mutant expressing lines, but apoptosis was rapidly induced by the addition of the ULK1‐dependent autophagy inhibitor MRT68921.

**FIGURE 7:**
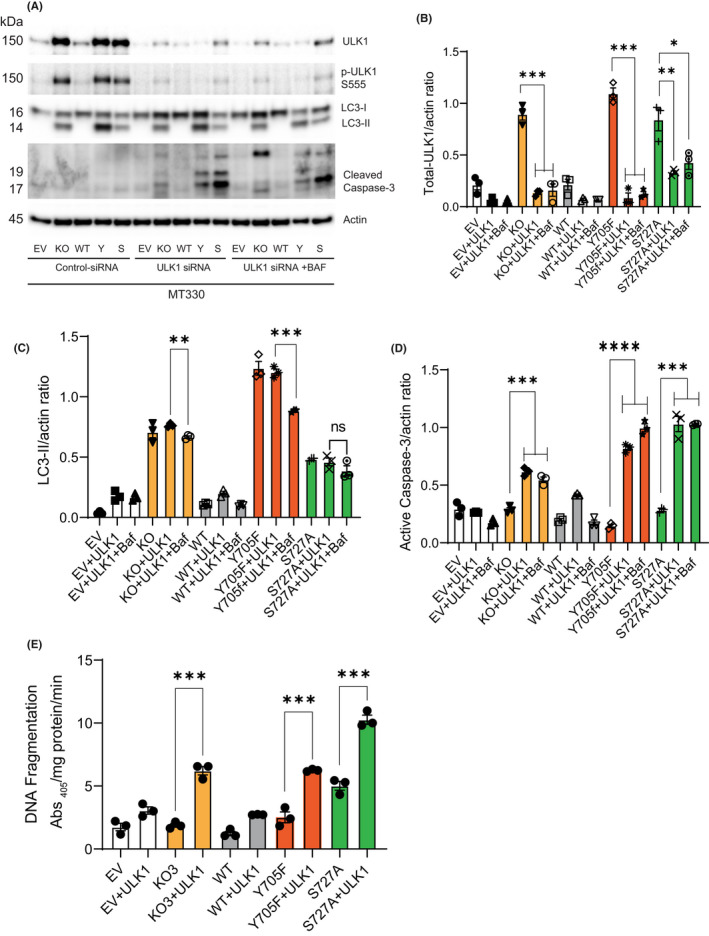
Knockdown of ULK1 expression blocks autophagy and induces apoptosis in STAT3‐KO and STAT3‐mutant expressing lines. (A) MT330 EV, STAT3‐KO, WT, Y705F and S727A mutant expressing cells were grown to confluence and treated with control or ULK1 siRNA followed by treatment with or without Baf for 3 h. ULK1 knockdown was verified by Western blotting with ULK1 and phospho‐ULK1, and cell lysates were analysed for LC3, cleaved caspase‐3 with β‐Actin as a loading control. Quantification of the ratio of (B) total‐ULK1 and Actin; (C) LC3‐II and Actin; (D) active caspase‐3 and Actin. (E) Apoptosis was measured by ELISA as in Figure 6F (mean SE, *n* = 3)

To further confirm the role of ULK1 in autophagy regulation, we knocked down ULK1 with siRNA in STAT3‐deleted MT330 cells and STAT3‐mutant expressing lines that contained high levels of ULK1 (Figure 1E). Control scrambled siRNA had no effect on ULK1 levels, but ULK1‐specific siRNA significantly decreased ULK1 levels in STAT3‐KO cells and cells expressing both Y705F and S727A mutants (Figure 7A,B). LC3‐II levels did not increase in ULK1 knockdown cells but significantly decreased in knockdown cells treated with Baf (Figure 7A,C). However, LC3‐II levels remained unchanged in ULK1 knockdown cells expressing the S727A mutant and treated with ULK1‐siRNA (Figure 7C), again demonstrating a key role of Y705 phosphorylation in the STAT3‐mediated suppression of autophagy via ULK1 signalling These studies suggest that autophagy induction by combining both STAT3 and ULK1 inhibition may be therapeutically beneficial for autophagy defective GBM cells.

To investigate whether ULK1 knockdown induces apoptosis, we measured caspase‐3 activation. Consistent with our observations involving inhibiting ULK1 activity by MRT68921 (Figure 6), ULK1 knockdown increased caspase‐3 activation and cell death in STAT3‐KO cells and cells expressing both phosphorylation‐defective mutants in the presence and absence of Baf (Figure 7A,D,E). However, caspase‐3 cleavage and cell death were considerably greater in cells expressing the S727A‐STAT3 mutant treated with ULK1‐siRNA.

**FIGURE 8:**
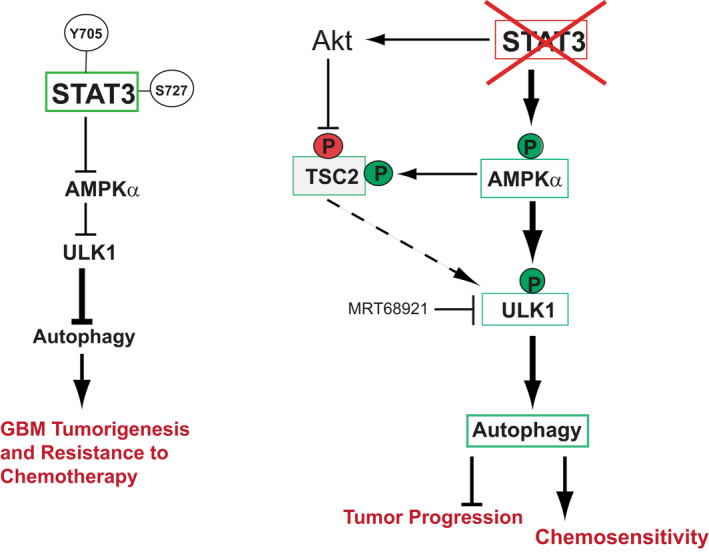
Schematic representation of the molecular cross talk inSTAT3‐dependent autophagy and chemoresistance in GBM. →, Activation; ⊥, inhibition; (P) in red indicates phosphorylation causing inhibition; and (P) in green indicates phosphorylation leading to activation. Boxes in green indicate protein activation, and boxes in red indicate inhibition

Based on our data, we propose a conceptual model (Figure 8, left) by which STAT3 represses autophagy and promotes GBM tumorigenesis. STAT3's post‐translational modifications (PTMs) are responsible for inhibiting autophagy in GBM cells. STAT3 PTMs inhibit AMPKα and ULK1 signalling in GBM cells, which in turn inhibit autophagy. This promotes GBM tumour formation and increases chemoresistance. Our data indicate that both Y705 and S727 phosphorylation are essential for autophagy suppression, and Y705 plays the predominant role in the inhibition of AMPKα/ULK1 signalling. Deleting STAT3 decreases p62 protein levels, increases AMPKα activity, and ULK1 gene expression and activity (Figure 8, right). These changes in cellular signalling trigger autophagy in GBM cells. Under basal conditions, mTOR is activated in GBM cells, but autophagy induction in STAT3‐KO cells is independent of the mTOR pathway. Activated AMPKα phosphorylates TSC2 at S1387, which keeps mTORC1 activity in check leading to autophagy. Because STAT3 deletion activates Akt at S473, which inhibits TCS2 via phosphorylation at T1462, resulting in basal mTORC1 activation, we speculate that baseline activation of mTORC1 in GBM cells could phosphorylate and inhibit ULK1 at S638. This is counteracted by extensive ULK1 phosphorylation at multiple sites causing its sustained activation and favouring its association with AMPKα. As a result, AMPKα directly phosphorylates ULK1 at S555 maintaining autophagy flux in STAT3‐KO cells.

STAT3 deletion also increases expression of the pro‐autophagy protein, Prom1,^34^ in GBM cells. These molecular events enhance autophagy flux and reduce GBM tumorigenesis. Inhibiting ULK1 signalling in STAT3‐KO cells validates the conceptual model described above. Inhibition of mTORC1 by Everolimus stimulates autophagy, whereas inhibition of ULK1 (by MTY68921 and siRNA) inhibits autophagy and induces apoptosis in STAT3‐KO cells. Together, these studies demonstrate that STAT3‐dependent suppression of autophagy is an essential contributor to GBM biology and that restoration of autophagy by a combined approach through STAT3 inhibition and AMPKα/ULK1 activation may be a novel approach to treat and overcome chemoresistance in GBM.

## 4 DISCUSSION

A key finding of these studies is that constitutive STAT3 phosphorylation suppresses autophagy in GBM cells. KO of STAT3 and the expression of STAT3 phosphorylation‐defective mutants in GBM cells increase autophagy via upregulation of p62/SQSTM1 degradation, LC3 conversion and lysosomal activity. Loss of STAT3 functional activity increases phosphorylation of AMPKα, TSC2 and ULK1 on multiple sites, which were reversed by expression of ectopic WT‐STAT3 in STAT3‐KO GBM cells. Expression of the Y705F and S727A mutants in STAT3‐KO LN229 and MT330 cells robustly increases autophagy, demonstrating that STAT3 function is indispensable for suppressing autophagy in GBM cells.

The role of autophagy in cancer is controversial because it has been reported to both promote and inhibit tumorigenesis.^40^ Autophagy confers drug resistance to radiotherapy and chemotherapy but also slows tumour progression.^41^ In contrast, rapid tumour growth in GBM and insufficient nutrient supply from the tumour vasculature contribute to activating autophagy and desensitizing tumour cells to chemotherapy.^42^ We found that basal autophagy was undetectable in MT330 GBM cells and that STAT3‐KO induced autophagy without altering mTOR activity in both MT330 and LN229 cells. Inhibiting mTOR in STAT3‐KO MT330 cells potentiated autophagy suggesting that combining STAT3 and mTOR inhibitors may improve GBM responses to treatment. Given these complex relationships, a better understanding of autophagy induction in response to STAT3 inhibition will benefit our understanding of GBM chemoresistance.

STAT3 undergoes serine and tyrosine phosphorylation that are molecular switches governing STAT3 activation and localization. Besides its well‐known Y705 phosphorylation, STAT3 is phosphorylated on S727, which regulates its mitochondrial localization.^43^ Cells expressing the Y705F mutant are viable and proliferate albeit at a slower rate compared to cells expressing WT‐STAT3. Other studies have demonstrated that the STAT3‐Y705F mutant can form dimers and the preformed unphosphorylated dimers were present in both stimulated and unstimulated cells.^44^ Other STAT3 PTMs include acetylation on K685 by CBP/p300, S‐glutathionylation by intracellular oxidative stress and trimethylation by EZH2.^45^ We found that STAT3 is constitutively phosphorylated on Y705 and S727 residues, acetylated on K685 and trimethylated on K180 (Figure S1), but these modifications were interdependent in GBM cells. STAT3‐KO completely abolished acetylation and trimethylation, and rescue of KO cells with WT‐STAT3 restored these PTMs. While expression of the STAT3 mutants in KO cells did not significantly alter STAT3 acetylation at K685, expression of the Y705F mutant increased STAT3 trimethylation, but the S727A mutant decreased STAT3 trimethylation, again suggesting a central role of STAT3 Y705 phosphorylation in regulating STAT3 function. STAT3‐S727 phosphorylation is dependent on STAT3‐Y705 phosphorylation, but Y705 phosphorylation is independent of S727 phosphorylation.^12^

STAT3 has been shown to regulate autophagy through several mechanisms.^46^ Nuclear STAT3 regulates autophagy through the transcriptional regulation of pro‐autophagy genes such as Beclin1 (BECN1) and anti‐ or pro‐autophagy modulating microRNAs.^46^, ^47^ Our qPCR analyses of autophagy genes demonstrate that STAT3 inhibits ULK1 expression but increases expression of p62 and BNIP3 genes. STAT3‐KO decreases BNIP3 and p62 protein levels but increases ULK1 expression in MT330 cells, showing a correlation between mRNA and protein levels. The BH3 domain‐containing protein, BNIP3, is regulated by STAT3 phosphorylation. BNIP3 expression is linked with induction of autophagy and requires upregulation of concanavalin‐induced JAK2/STAT3 signalling in GBM cells.^48^ In contrast, we found that STAT3‐deletion and expression of the phosphorylation‐inactive STAT3 mutants significantly decreased BNIP3 gene expression and protein levels showing that BNIP3 downregulation correlates with autophagy induction in GBM cells. These differences support the notion that BNIP3 plays diverse roles in GBM autophagy regulation, and these roles may be stimuli and cell context dependent.

Since enhanced autophagy results in p62 degradation and upregulation of Prom1 expression, we expected that induction of autophagy flux by STAT3 deletion would lead to enhanced degradation of p62 and increase Prom1 protein levels. Supporting this, STAT3‐KO and Y705 mutant expressing cells showed reduced p62 protein levels by activation of autophagy flux. Control and WT‐STAT3 expressing STAT3‐KO cells had reduced autophagy flux and impaired autolysosomal p62 degradation in GBM cells. STAT3‐KO and mutant expressing cells show upregulation of Prom1 protein levels, confirming autophagy induction. In addition, our results showed for the first time that p62 gene expression was significantly downregulated in these lines demonstrating that p62 is transcriptionally regulated by STAT3. Reconstitution of STAT3‐KO cells with WT‐STAT3 restored p62 gene expression confirming that STAT3 regulates p62 at the transcriptional level in MT330 cells. In contrast, p62 gene expression was unaltered in STAT3‐KO and phosphorylation‐defective mutant expressing LN229 lines. However, p62 protein levels were still reduced in these cells, suggesting transcription‐independent but autophagy‐dependent regulation of p62 in LN229 cells.

AMPKα maintains energy homeostasis and plays an important role in autophagy induction. AMPKα negatively regulates mTORC1 through TSC2, activates ULK1 Ser555 and Beclin1 Thr388 phosphorylation, all of which initiate autophagy.^49^ ULK1 plays a central role in autophagy by promoting fusion of autophagosomes with lysosomes and phosphorylating multiple autophagy‐related targets including Beclin1 and ATG101.^50^ Our data show that the AMPKα/ULK1 signalling axis regulates STAT3‐dependent autophagy in GBM cells. AMPKα and ULK1 phosphorylation is low in control EV GBM cells but was markedly increased in both STAT3‐KO MT330 cells and cells expressing STAT3 phosphodeficient mutants. In LN229 and MT330 cells, STAT3‐KO and Y705F mutant expressing cells showed elevated AMPKα activity. However, expression of the S727A mutant had no effect on AMPKα activity in LN229 cells but did so in MT330 cells, which suggests that Y705 phosphorylation regulates autophagy via AMPKα signalling in MT330 cells but not in LN229 cells. Rescue of KO cells with WT‐STAT3 abolished AMPKα T172 phosphorylation confirming STAT3's role in repressing AMPKα signalling in both LN229 and MT330 GBM cell lines. Control EV MT330 cells were resistant to autophagy induction in response to mTOR inhibition (using Everolimus). These results demonstrate that the attenuating effect of STAT3 on autophagy induction primarily depends on Y705 phosphorylation and its ability to inhibit AMPKα signalling pathway. Furthermore, our results show STAT3 is a novel suppressor of ULK1 in MT330 cells. Knockout of STAT3 dramatically increases ULK1 protein and gene expression in MT330 cells, which were blocked by expression of WT‐STAT3. This is consistent with earlier studies showing STAT1 as a transcriptional suppressor of autophagy through inhibition of ULK1 protein and mRNA levels.^51^ In LN229 cells, STAT3‐KO had no effect on ULK1 mRNA and protein levels but increased ULK1 activity, demonstrating that enhancement of ULK1 activity is transcription independent. Our studies highlight the function of various phosphorylation‐sites in STAT3 mutants, and our data unambiguously demonstrate that Y705F and S727A mutants differentially regulate AMPKα and ULK1 signalling to activate autophagy in GBM lines. In this context, our data also support the cellular and molecular heterogeneity seen between LN229 and MT330 GBM lines. This may account for the observed variability in AMPKα signalling in response to STAT3 deletion and expression of STAT3 mutants.

AMPKα activation inhibits mTORC1, which leads to autophagy. In addition to regulating mTORC1, AMPKα activation inhibits tumour cell growth by phosphorylating TSC2 on S1387, which in turn inhibits mTORC1 leading to autophagy activation.^52^ Our data demonstrate that cells lacking STAT3 and cells expressing STAT3 mutants have higher amounts of TSC2 T1462 and S1387 phosphorylation. The regulation of TSC2 and mTORC1 by AMPKα has special implications in autophagy regulation. While TSC2 T1462 phosphorylation inhibits its activity leading to mTORC1 activation,^53^ AMPKα inhibits mTORC1 in part by phosphorylating and activating TSC2 on S1387.^54^ ULK1 S555 phosphorylation is mediated through AMPKα and indicates autophagy activation, whereas other ULK1 sites are targeted by mTOR to inhibit autophagy.^28^ Conversely, ULK1 inhibits the kinase activity of mTORC1 to stimulate autophagy.^55^ We find that AMPKα is activated in STAT3‐KO and STAT3‐KO GBM cells expressing phosphorylation‐defective STAT3 mutants, which suggests that AMPKα directly phosphorylates ULK1 on several sites required to sustain ULK1 activation. This mechanism of ULK1 activation is sufficient to inhibit mTORC1 and activate autophagy. Future studies are needed to further define how STAT3 regulates AMPKα signalling, but our data suggest that activated AMPKα induces autophagy in STAT3‐KO cells through TSC2 to directly activate ULK1.

Consistent with STAT3 suppressing autophagy through inhibition of AMPKα/ULKI signalling, we found that inhibiting ULK1 activity by MRT68921 or ULK1 protein knockdown by siRNA decreased autophagy, and sensitized STAT3‐KO and STAT3‐phosphodeficient mutant expressing lines to caspase‐3‐dependent apoptosis. These results suggest that autophagy is cytoprotective in STAT3‐KO cells. Thus, approaches directed at inhibiting ULK1 may inhibit autophagy through downstream blockade of ULK1‐Atg14‐Beclin1 signalling and consequently lead to GBM cell death. To our knowledge, this is the first example of the involvement of ULK1 signalling in the regulation of STAT3‐dependent autophagy/apoptosis and highlight the targeting of both STAT3 and ULK1 as a potential novel therapeutic approach for GBM treatment.

## AUTHOR CONTRIBUTIONS

**Sujoy Bhattacharya:** Conceptualization (lead); data curation (lead); formal analysis (lead); investigation (lead); methodology (lead); project administration (lead); validation (equal); writing – original draft (lead); writing – review and editing (supporting). **Jinggang Yin:** Data curation (supporting); formal analysis (supporting); investigation (supporting); methodology (supporting). **Chuanhe Yang:** Data curation (supporting); formal analysis (supporting); investigation (supporting). **Yinan Wang:** Data curation (supporting); investigation (supporting); methodology (supporting). **Michelle Sims:** Data curation (supporting); methodology (supporting). **Lawrence M. Pfeffer:** Conceptualization (supporting); data curation (supporting); investigation (lead); project administration (supporting); resources (supporting); supervision (supporting); validation (supporting); writing – original draft (supporting); writing – review and editing (equal). **Edward Chaum:** Conceptualization (supporting); formal analysis (supporting); funding acquisition (lead); investigation (supporting); project administration (lead); resources (lead); supervision (lead); writing – original draft (supporting); writing – review and editing (supporting).

## CONFLICT OF INTEREST

The authors confirm that there are no conflicts of interest.

## Supporting information


Figure S1

## Data Availability

The data that support the findings of this study and the STAT3‐KO and mutant expressing GBM cells generated during and/or analysed during the current study are available from the corresponding author on reasonable request.
