# Supplementary material for: STAT3 suppresses the AMPKα/ULK1‐dependent induction of autophagy in glioblastoma cells
Source: J Cell Mol Med. 2022 Jun 6;26(14):3873–90. doi: 10.1111/jcmm.17421 (PMC9279602; doi:10.1111/jcmm.17421)
Supplement: Supplementary file 1 — Figure S1 [file JCMM-26-3873-s001.pdf]

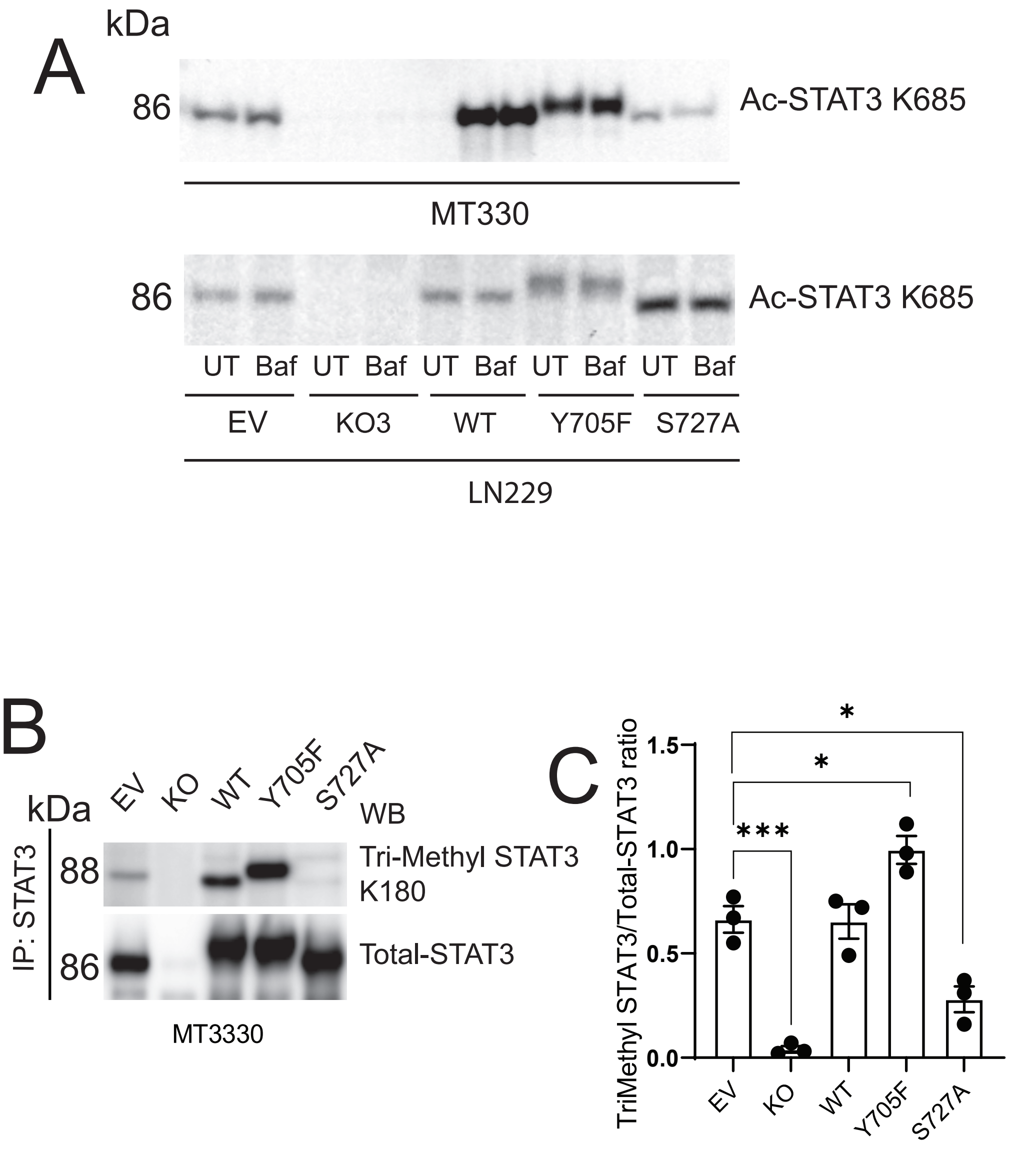

Supplemental Fig. 1- Posttranslational modifications (acetylation and tri-methylation) of STAT3 in GBM cells.

(A) Control EV MT330 and LN229 cells, STAT3-KO, and KO-cells reconstituted with WT and phosphoryation-defective Y705F-STAT3 and S727A-STAT3 mutants were exposed to bafilomycin (Baf, 100nM) for 3h. Cell lystaes were immunoblotted with Acetyl (Ac)-STAT3 K685 antibody.

(B) Cells lysates were immunoprecipitated (IP) with STAT3 and immunoprecipitates were western blotted (WB) with Tri-methyl-K180 STAT3. Membranes were stripped and probed for total-STAT3.

(C) Quantification of Trimethyl- to total-STAT3.
